# Semantic versus perceptual priming: dissecting their impact on intuitive judgments of semantic coherence

**DOI:** 10.3389/fpsyg.2024.1406811

**Published:** 2024-06-25

**Authors:** Joanna Sweklej, Robert Balas

**Affiliations:** ^1^Department of Psychology, SWPS University, Warsaw, Poland; ^2^Institute of Psychology, Polish Academy of Sciences, Warsaw, Poland

**Keywords:** intuition, judgments of semantic coherence, semantic priming, perceptual priming, confidence

## Abstract

This research explores the mechanisms underlying the intuitive processing of semantic coherence, focusing on the effects of semantic and perceptual priming on semantic coherence detection. Two studies examined how these priming types influence individuals’ abilities to discern semantic incoherence. In Study 1, we used solutions to semantically coherent triads as primes, finding that such priming significantly improves participants’ accuracy and confidence in identifying incoherent elements within word tetrads. These results corroborate the hypothesis that intuitive judgments in linguistic tasks are closely tied to the processing fluency elicited by semantic connections. In Study 2, we show that perceptual priming does not significantly enhance accuracy, albeit it does increase the confidence with which individuals make their judgments. Distinct effects of semantic and perceptual priming on intuitive judgments highlight the complex interplay between processing fluency and affect in shaping intuitive judgments of semantic coherence. We discuss the nuanced roles of semantic and perceptual factors in influencing the accuracy and confidence of intuitive decisions.

## Introduction

Intuition has long been a subject of interest in psychology and the public sphere due to its prevalence in everyday life and its counterintuitive (*sic*!) association with making decisions without conscious reasoning. To date, it has been many times shown that intuitive decision-making involves a blend of fast, automatic processes and slower, conscious processes, influenced by various cognitive factors such as executive functions, affect, and the use of heuristics (Bolte and Goschke, 2005). Intuition also involves judging stimulus properties based on activated but not consciously retrieved information from memory (Topolinski and Strack, 2009a). Furthermore, it shares similarities with implicit memory processes due to its experiential basis (Zander et al., 2016). The research on intuition aims at disentangling automatic and conscious processes, affective states, and cognitive mechanisms all play a role in guiding intuitive decision-making, judgment, and evaluations. Understanding how intuition operates provided insights into human cognition and behavior.

This understanding of intuition fits well within the framework of dual-process theories of judgment, which propose that human cognition operates via two distinct systems: Type 1 (intuitive, fast, automatic) and Type 2 (deliberative, slow, analytical). According to dual-process theories, including those articulated by Kahneman (2013), Type 1 processes align closely with the automatic, experiential aspects of intuition, characterized by rapid and effortless judgments that often rely on heuristics and affective responses. In contrast, Type 2 processes involve more deliberate and effortful reasoning, which can override initial intuitive responses when deeper cognitive engagement is required. This interplay between the two systems underscores the complexity of intuitive decision-making, as intuitive judgments (Type 1) can be validated or adjusted by reflective processes (Type 2), ensuring a more comprehensive approach to understanding human cognition and behavior (Evans, 2008; Evans and Stanovich, 2013; Kahneman, 2013; Gawronski et al., 2014).

It is worth mentioning that dual-process theories are a class of theoretical assumptions, and different variations of these theories adopt them (see Pyszczynski et al., 1999; Strack and Deutsch, 2004). One example of this more specific application of dual processes is the Associative-Propositional Evaluation (APE) model (Gawronski and Bodenhausen, 2011). This model posits that attitude formation involves two distinct mental processes: associative processes, which are quick and intuitive, and propositional processes, which are more deliberate and effortful. According to the APE model, automatic evaluation is primarily driven by activating evaluative associations, while deliberate evaluation involves considering these associations as valid evaluative evidence (Moran and Bar-Anan, 2013). The APE model has been applied in various contexts, such as understanding implicit bias recognition and management and investigating the influence of faith, intuition, and method of attitude formation on implicit-explicit brand attitude relationships. For our purposes, the APE model represents two different routes to intuitively detect semantic coherence: associative (through semantic associations in memory) and propositional (through reflective evaluation of semantic relations). We assume that both associative and propositional processes are involved in responses in a semantic coherence task.

The semantic coherence task, developed by Bowers et al. (1990), is a valuable model and research tool for studying intuitive responding (Bolte and Goschke, 2005). This task is based on the Remote Associates Test – RAT (Mednick and Mednick, 1967) and has shown that individuals often struggle to provide a common associate for a semantically coherent word triad. However, they are surprisingly accurate in determining whether a given word triad has a common associate (Bowers et al., 1990; Topolinski and Strack, 2009a; Balas et al., 2012). Research by Topolinski and Strack (2009a,b), Balas et al. (2012), and Sweklej et al. (2015) suggests that processing fluency and positive affect are critical mechanisms in intuitive decision-making when assessing semantic relations of word triads (Zander et al., 2017). This approach aligns with the findings that inducing a positive mood can enhance intuitive coherence judgments (Bolte et al., 2003). Their work sheds light on how fluency and affect play crucial roles in determining intuitive judgments of semantic coherence. In essence, the intuitive semantic coherence task provides insights into how individuals navigate semantic relationships intuitively by highlighting the significance of emotional factors, processing fluency, and affect in shaping intuitive coherence judgments.

A significant amount of research expanded on Bowers et al.’s (1990) work on intuitive judgments of semantic coherence (Topolinski and Strack, 2009a). The mechanism that is assumed involves increased processing fluency of semantically coherent material. Semantically coherent concepts are interlinked in memory through semantic associations, and once a given concept is activated, the activation spreads to semantically related concepts (Undorf and Zander, 2017). Therefore, it is easier and more fluent to process semantically related material. That processing fluency generates, in turn, a subtle transient positive affective response that, in turn, guides participants’ responses (Reber et al., 2004; Winkielman et al., 2006; Topolinski et al., 2009). Therefore, the supposed and experimentally tested mechanism assumes increased processing fluency of semantically coherent material, where semantically related concepts are interlinked in memory and activate positive affective responses to guide intuitive judgments of semantic coherence.

The assumption of the processing fluency model is that participants are sensitive to coherence because they can detect changes in processing fluency. Sweklej et al. (2014) explored this idea using semantically coherent triads embedded in tetrads – a set of four words consisting of coherent triads plus an additional unrelated word. The key finding of their research was that participants were able to detect incoherent words within tetrads, indicating that they were sensitive to changes in processing fluency that could signal an odd item within semantically coherent triads (Sweklej et al., 2014). The novelty of these findings was that changes in processing fluency can impact individuals’ ability to detect inconsistencies within coherent sets of information. Demonstrating that participants can identify incongruities within semantically coherent triads further confirmed the importance of processing fluency in cognitive processes related to coherence judgments.

The current research aims to expand Sweklej et al. (2014) findings and investigate the impact of different types of priming, specifically semantic and perceptual priming, on participants’ ability to detect semantic incoherence. Semantic priming involves briefly presenting a semantic prime that activates a network of semantically related concepts to enhance processing fluency (De Wit and Kinoshita, 2015). Perceptual priming employs the degraded presentation of a stimulus that increases processing fluency via perceptual facilitation (Susser et al., 2016).^1^ Processing fluency can stem from associative activation of semantic relations in memory or easier processing of more perceptually salient stimuli. Both mechanisms can result in more fluent processing, which has been shown to affect intuitive responses. However, we expect that the activation of semantic associations through semantic priming will influence intuitive judgments more due to the semantic nature of the task.

In contrast, manipulating processing fluency through perceptual priming has a lower probability of increasing intuitive judgments of semantic coherence since it does not directly address semantic association. Therefore, we expect differences in the accuracy of intuitive judgments between semantic and perceptual manipulations. This expectation aligns with dual-process theories, highlighting the interplay between intuitive and deliberative processes in shaping judgments. In two studies, we seek to discern whether the methods mentioned earlier yield varying effects on semantic incoherence detection. We hope to contribute to understanding how changes in this fluency affect intuitive judgments of semantic coherence.

## Study 1

This study aims to investigate the cognitive processing underlying linguistic intuition by examining the effects of masked primes on tetrad puzzle-solving tasks. Specifically, the research seeks to explore how the presentation of solvable and unsolvable tetrads, primed with solution words or nonwords, influences participants’ ability to identify outlier words, confidence in decision-making, and ability to propose solutions.

### Method

The sample size was determined by G*Power software (Faul et al., 2007) based on our previous data (Sweklej et al., 2014) acquired in a similar paradigm. We calculated a sample size assuming a one-sample *t*-test against a 0.25 chance probability of correctly identifying the unrelated item within a word tetrad. Assuming a moderate effect size of Cohen’s *d* = 0.5 with a power of 0.95, the required sample size turned out to be 45. We set the desired sample size to 100 participants due to differences in materials used by Sweklej et al. (2014), expected exclusions due to an awareness check, and the possibility of effect size being lower than moderate.

#### Participants

A total of 113 participants (65 men and 48 women) were recruited via Prolific for this study. The age of participants ranged from 22 to 40 years, with a mean of 31.68 years (*SD* = 4.98). All participants were native English speakers. They volunteered for the study and were compensated for their time.

#### Materials

A total of 60 tetrads used in this study were constructed as follows. First, 40 solvable triads were selected from the (Bowden and Jung-Beeman, 2003) set of remote associate problems. Each selected triad was matched with a non-associated word randomly sampled from the above set. Thus, each solvable tetrad consisted of a triad and an unrelated fourth word. Second, another 40 tetrads were assembled by randomly sampling words from Bowden and Jung-Beeman (2003). Finally, two sets of primes were selected. The first set of primes (*N* = 40) consisted of solution words to the triads used in solvable tetrads. The second set of primes included 40 two-syllable nonwords. The materials used across Studies 1 and 2 are presented in Appendix 1.

#### Procedure

After informed consent and detailed instructions, participants were shown 60 tetrad trials, each displaying four words for 5 s. These trials were divided into 20 solvable tetrads with solution word primes, 20 solvable tetrads with nonword primes, and 20 unsolvable tetrads with nonword primes. The unsolvable tetrads served as filler trials. Primes were masked and briefly presented for 80 milliseconds before each tetrad. Tetrads were randomly selected from a stimuli pool and presented randomly to each participant.

**Figure 1:**
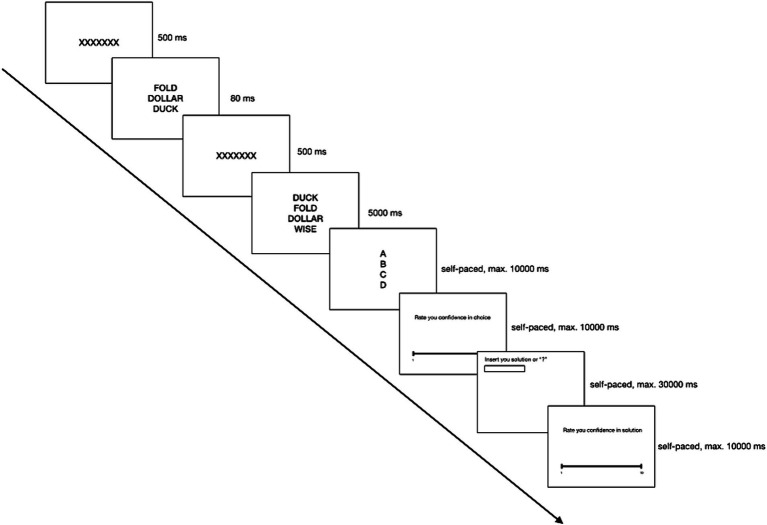
A single trial order and timing in Study 1.

Each trial required participants to identify the non-related word via keypress (A, B, C, or D) and rate their choice certainty on a Likert scale from 1 (utter uncertainty) to 10 (absolute certainty). Participants were then instructed to write a proposed solution word for the coherent triad or indicate uncertainty with a question mark, subsequently rating their solution certainty using the same scale. A single trial order and timing are illustrated in Figure 1.

An attention check followed the tetrad trials. Participants were explicitly instructed NOT to mention any sports activities they practiced and given a list of some sports activities to choose from. A failure to comply with the explicit instruction resulted in excluding their data from further analysis. This check ensured attentiveness and compliance with study protocols.

### Results

Five out of 113 participants failed the attention check and were excluded from analyses

#### Accuracy of choices

The accuracy of choices, as well as any further dependent variables, were analyzed with a linear mixed-effects model rather than traditional repeated measures methods such as analysis of variance (ANOVA) because the former allows for better handling of dependencies in repeated measures data (Misangyi et al., 2006).^2^ Participants demonstrated significantly higher accuracy in identifying the unrelated word in solvable tetrads primed with solution words (*M* = 0.605, *SE* = 0.019) compared to those primed with nonwords (*M* = 0.464, *SE* = 0.011), *F*(1, 111.48) = 46.763, *p* < 0.001. The model fit was singular. Therefore, we eliminated its intercept to leave subject variability associated with a fixed factor.

D-prime scores for each participant were calculated based on signal detection theory principles, which quantify participants’ ability to distinguish correct from incorrect responses. The hit rate was computed as the proportion of trials in which participants correctly identified the target word, while the false alarm rate was defined as the average rate of incorrectly choosing any of the three distractor words. These rates were converted to z-scores using the inverse cumulative distribution function of the standard normal distribution. The d-prime was then calculated as the difference between the z-scores of the hit rate and the false alarm rate, with continuity corrections applied to adjust hit rates of 1.0 or 0 and false alarm rates of 1.0 or 0 to ensure all calculated values remained finite and computationally valid.

The linear mixed model was fit using REML (Restricted Maximum Likelihood) with d-prime as the dependent variable. The model included type of priming (solution primes vs. nonword primes) (as a fixed effect and subject) as a random intercept. The REML criterion at convergence was 460.4, indicating the model’s fit. The random effects structure reveals that variability in *d-prime* scores between participants (intercept variance) is relatively small (variance = 0.05376, SD = 0.2319), suggesting modest between-subject variability after controlling for the type of priming effects. The fixed effects analysis shows a significant effect of the type of priming condition on *d-prime* scores. The average *d-prime* score for the reference group (nonword primes) is significantly positive (Estimate = 0.82224, SE = 0.07003, *t*(205.76) = 11.741, *p* < 0.001). This suggests a strong ability to distinguish between correct and incorrect word choices in the nonword priming condition. Compared to the nonword priming condition, participants in the solution priming condition demonstrated significantly higher *d-prime* scores (Estimate = 0.63650, SE = 0.09373, *t*(104.00) = 6.791, *p* < 0.001). This result indicates a better discriminative performance in the solution priming condition.

#### Confidence in choices

An LME analysis of within-subject effects demonstrated a significant Tetrad Type effect, *F*(2, 98) = 50.982, *p* < 0.001. For unsolvable tetrads, the mean confidence was 3.528 (*SE* = 0.186, CI = 3.164–3.893); for solvable tetrads primed with nonwords, the mean was slightly higher at 3.755 (*SE* = 0.174, CI = 3.415–4.095); and for solvable tetrads primed with solution words, participants exhibited the highest confidence with a mean of 5.648 (*SE* = 0.174, CI = 5.298–5.999).

#### Accuracy of solutions

The analysis of participants’ responses when prompted to propose solutions for tetrads revealed that a mean proportion of 54.12% of the responses were marked as “do not know” (a question mark). The mean proportion of accuracy of proposed solutions to solvable tetrads was 12.54%, which prevented the comparison of these accuracies between types of tetrads due to low power.

#### Confidence in solutions

To analyze participants’ confidence in proposed solutions, we filtered out responses that were either “do not know” or missing data from the database. The resulting pool of proposed solutions revealed only 12 individual participants. Therefore, we did not analyze their confidence in the proposed solutions due to low power.

#### Accuracy and confidence in intuitive choices

As we can see above, participants declared no knowledge (or even supposition) of solutions in most trials. We deem those cases intuitive because of a lack of awareness of solutions. To determine how priming influences the accuracy and confidence of intuitive choices, we first compared mean choice accuracy between tetrads primed with nonwords and those primed with solution words. The paired samples *t*-test indicated significantly higher accuracy for tetrads primed with solution words (*M* = 0.575, *SD* = 0.185) compared to nonword primed tetrads (*M* = 0.462, *SD* = 0.111), *t*(96) = −5.339, *p* < 0.001, with a moderate effect size (Cohen’s *d* = −0.542, *SE* = 0.144).

Further, we explored mean confidence in choices without explicit knowledge of solutions across different tetrad types using an LMM. The model included random intercepts for subjects, suggesting that variability in the baseline response across subjects is moderately high (Variance = 2.713, SD = 1.647). The residual variance within subjects across measurements was also notable (Variance = 2.983, SD = 1.727). This indicates substantial individual differences in response levels and notable variability in responses within subjects. The model included random intercepts for subjects, suggesting that variability in the baseline response across subjects is moderately high (Variance = 2.713, SD = 1.647). The residual variance within subjects across measurements was also notable (Variance = 2.983, SD = 1.727). This indicates substantial individual differences in response levels and notable variability in responses within subjects. The estimated average response for the incoherent condition is highly significant and positive (*β* = 2.973, SE = 0.169, *t*(111.7) = 17.598, *p* < 0.001). This suggests a strong baseline response level in the incoherent condition. The response in the nonword priming condition does not significantly differ from the baseline condition (*β* = −0.0047, SE = 0.0692, *t*(3113) = −0.068, *p* = 0.946). This indicates that the nonword condition does not affect the response level compared to the baseline incoherent condition. In contrast, the solution prime condition shows a significant increase in response (*β* = 0.6598, SE = 0.0858, *t*(3130) = 7.689, *p* < 0.001). This suggests that the solution prime condition significantly enhances the response level compared to the baseline condition. The estimated marginal means for baseline, nonword prime, and solution conditions were 2.97, 2.97, and 3.63, respectively.

**Figure 2:**
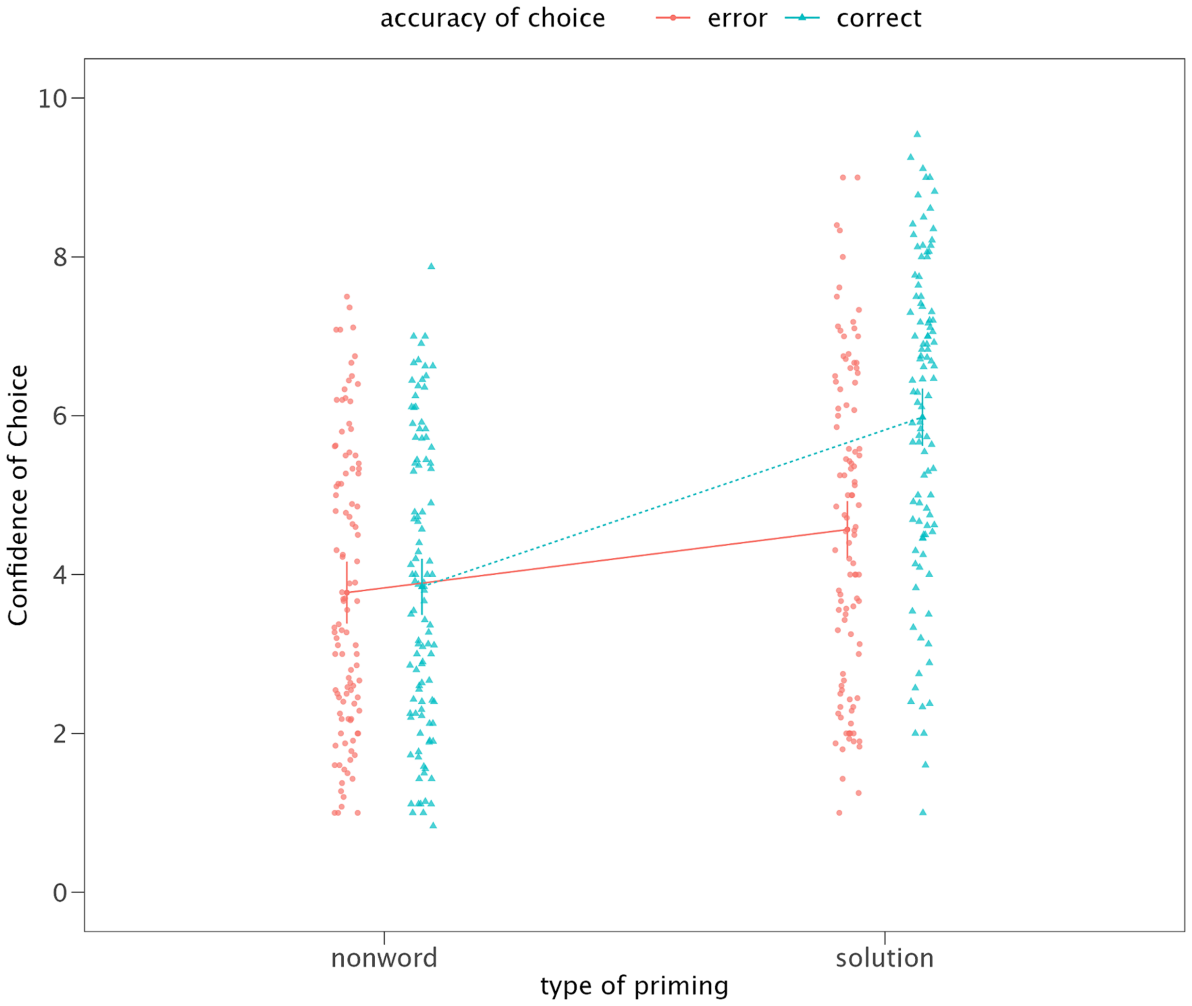
Mean confidence in intuitive choices depending on the type of priming and choice accuracy. Whiskers represent 95% CI.

The LMM that assessed the impact of choice accuracy and type of priming on confidence in choices was fit using restricted maximum likelihood (REML). The results from the fixed effects model indicate significant main effects for both the type of priming and the accuracy of choice, as well as a significant interaction between these two factors. Specifically, the type of priming had a significant effect on the mean response [*F*(1, 104) = 84.533, *p* < 0.001], as did the accuracy of choice [*F*(1, 104) = 30.817, *p* < 0.001]. Furthermore, the interaction between type of priming and accuracy of choice also showed a significant effect [*F*(1, 104) = 45.979, *p* < 0.001 – see Figure 2]. Variance components indicated considerable variability at the subject level (Variance = 1.929, SD = 1.389) and within the residual errors (Variance = 1.027, SD = 1.013). This implies significant individual differences in baseline response levels across subjects and variability in responses not explained by the fixed effects.

### Discussion

The findings from Study 1 offer insights into the cognitive underpinnings of linguistic intuition and the role of semantic priming in judgments of incoherence. The enhanced performance associated with solution word primes underscores the profound impact of semantic coherence on the processing fluency of participants, affirming the hypothesis that intuitive judgments in linguistic tasks are intricately tied to the processing fluency elicited by semantic priming. The high frequency of “do not know” responses suggests that participants found it difficult to get insight into the nature of semantic coherence. However, they appeared to detect incoherence of one word quite accurately when semantic coherence was enhanced due to brief exposure of solution words. These results suggest that the effectiveness of intuition in linguistic tasks is contingent upon the alignment between semantic cues and processing fluency. It also emphasizes the critical role of semantic priming in enhancing both the accuracy and confidence of intuitive decisions.

Moreover, the d-prime analysis suggests significant differences in discriminative performance (measured by *d-prime*) across the two conditions examined. Participants tend to perform better in the solution priming condition than in the nonword priming condition. The random effects indicate that while there is some variability in scores across individuals, this variability is relatively small compared to the fixed effect of the type of priming.

## Study 2

As argued before, two theoretically independent sources of processing fluency might exist. Study 1 explored semantic priming as a source of increased processing fluency and its consequences for intuitive judgments of semantic incoherence. In Study 2, we aimed to employ perceptual priming as an alternative source of fluency to see whether it aligns with the processing of semantic coherence and enables intuitive detection of incoherence.

### Method

The sample size was determined exactly as in Study 1

#### Participants

A total of 110 participants (41 men and 69 women) volunteered for the study via the Prolific online platform for monetary compensation. The age range of participants spanned from 18 to 40 years (*M* = 32.43, *SD* = 4.87). All participants were English native speakers.

#### Materials

In Study 2, the construction of materials followed a similar framework to that of Study 1, with specific modifications to explore the potential impact of perceptual priming. The study used the same set of 60 tetrads, comprising both solvable and unsolvable configurations, derived from the same set taken from Bowden and Jung-Beeman (2003). Two distinct types of perceptual primes were employed for solvable tetrads: one consisted of semantically coherent triads directly related to the given tetrad. At the same time, the other featured a random set of three words with no semantic relation to the tetrad. Conversely, unsolvable tetrads were uniformly primed with a randomly assembled set of three words.

#### Procedure

**Figure 3:**
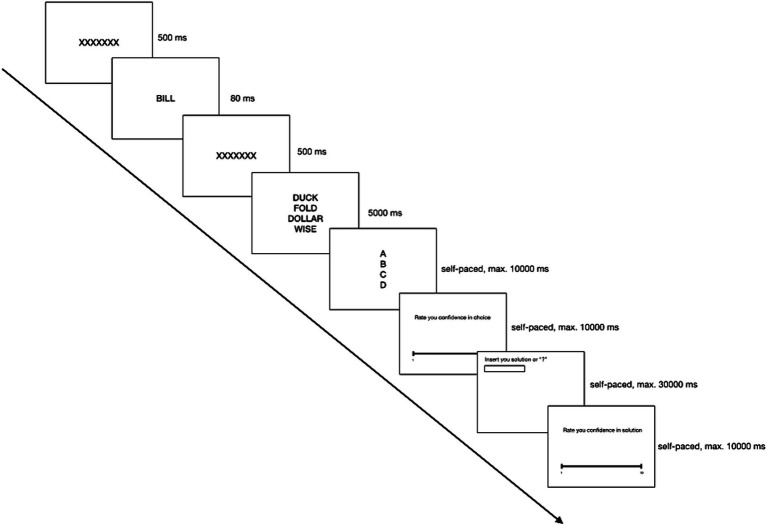
A single trial order and timing in Study 2.

The procedure for Study 2 mirrored that of Study 1, with the primary exception being the shift from semantic priming to perceptual priming. The perceptual primes were presented for 80 milliseconds and backward and forward masked as in Study 1. The tetrads were shown in a random order for 5 s. Each trial required participants to identify the unrelated word by pressing the corresponding key (A, B, C, or D) and to rate their certainty in their choice on a Likert scale ranging from 1 (utter uncertainty) to 10 (absolute certainty). Subsequently, participants were prompted to propose a solution word for the coherent triad or to indicate a “do not know” answer with a question mark, then rating their certainty in the proposed solution using the same scale (see Figure 3). An attention check, identical to that in Study 1, concluded the experiment.

### Results

Six out of 110 participants failed the attention check and were excluded from further analyses.

#### Accuracy of choices

The linear mixed model analysis was employed to examine the influence of block type on response accuracy while accounting for individual differences through subject-level random effects. Utilizing restricted maximum likelihood for estimation, the model converged with a REML criterion of 5332.2, indicating a satisfactory fit to the data.

In the random effects structure, the variability attributed to individual differences among subjects was relatively small yet significant, with a standard deviation of approximately 0.09446 for the random intercepts. This suggests modest variations in baseline accuracy across individuals. The residual variability was more pronounced, evidenced by a standard deviation of 0.45872, indicating substantial differences in accuracy that were not explained by the block type alone.

Regarding fixed effects, the baseline accuracy, which corresponds to the random priming condition, was significantly positive at 0.31765 with a standard error of 0.01381, reflecting a moderate level of accuracy (*M* = 0.318, SE = 0.0138, CI = 0.291–0.345). The coefficient for the perceptual priming condition indicated an increase in accuracy compared to the baseline (*M* = 0.331, SE = 0.0138, CI = 0.304–0.358), but this was not statistically significant, with an estimate of 0.01373 and a standard error of 0.01436. The *t*-value of 0.956 further underscores the lack of a significant impact of this priming on accuracy. Additionally, the negative correlation of −0.520 between the intercept and the effect of perceptual priming suggests that the potential influences of priming are less pronounced in subjects or contexts with inherently higher accuracy levels.

Similar to Study 1, we calculated d-prime scores for each participant to show possible differences in sensitivity related to perceptual priming. The LMM analysis of d-prime scores was run to assess the effects of perceptual priming on participants’ discriminative sensitivity in identifying correct versus incorrect stimuli. The model included random intercepts for subjects to account for individual differences and achieved a REML criterion of 405.3 upon convergence. This indicates an adequate fit of the model to the data. Regarding random effects, the analysis highlighted significant variability among subjects, with a standard deviation of 0.4464 for the subject-specific intercepts. This substantial variability suggests that individual differences significantly influence baseline discriminative ability. The residual variance, with a standard deviation of 0.4724, indicates additional variability in d-prime scores not captured by the perceptual priming or individual differences alone.

The fixed effects analysis showed that the baseline d-prime score was significantly positive at 0.208, with a standard error of 0.063, yielding a *t*-value of 3.323. This result suggests a moderate level of discriminative sensitivity under standard conditions. The influence of perceptual priming was relatively small, with an estimate of 0.062 and a standard error of 0.064, resulting in a *t*-value of 0.960. This indicates that the difference in discriminative sensitivity associated with priming was not statistically significant compared to the baseline. The correlation of −0.514 between the intercept and the effect of perceptual priming suggests a moderate inverse relationship between the baseline discriminative sensitivity and the effect of this block type. This could indicate that any potential influences of perceptual priming are less pronounced in subjects or contexts with inherently higher discriminative abilities.

#### Confidence in choices

The linear mixed model was utilized to investigate the impact of the type of priming on confidence in choices while accounting for individual differences across subjects. This model was fitted using restricted maximum likelihood estimation, as reflected in the REML criterion at convergence of 24913.3. The model’s random effects show considerable variability across subjects, with a standard deviation of 1.584 for the random intercepts, which indicates notable individual differences in baseline response levels. Additionally, the residual variability, with a standard deviation of 1.990, suggests significant differences in response that the model’s fixed effects do not explain.

Regarding fixed effects, the estimated intercept, which can be interpreted as the baseline confidence, was substantially high at 3.334 with a standard error of 0.163, yielding a *t*-value of 20.415, indicating a statistically significant effect. The random priming condition positively impacted the confidence with an estimate of 0.200 and a standard error of 0.064, leading to a *t*-value of 3.127. Similarly, the perceptual priming condition demonstrated a more pronounced effect, with an estimate of 0.449 and a standard error of 0.064, which resulted in a *t*-value of 7.034. Both these effects were statistically significant and suggested that different priming conditions have distinct influences on participants’ confidence, with perceptual priming showing the most substantial impact. The correlation matrix for fixed effects indicated a moderate positive correlation between the effects of the two priming conditions, suggesting that their influences on confidence are not entirely independent.

#### Accuracy of solutions

Like Study 1, participants responded “do not know” when asked to propose solutions for tetrads in 52.24% of the cases. The mean proportion of accuracy of proposed solutions to solvable tetrads was 9.34%, which prevented the comparison of these accuracies between types of tetrads due to low power.

#### Confidence in solutions

As in Study 1, we filtered out responses that were either “do not know” or missing data from the database to look for the differences in how confident participants were in the solutions they proposed. The resulting pool of proposed solutions revealed only five individual participants. Thus, we skip this analysis due to low power.

#### Accuracy and confidence in intuitive choices

The linear mixed model was applied to investigate the impact of perceptual priming on participants’ accuracy in choices made without explicit knowledge about actual solutions. Analysis was conducted using restricted maximum likelihood (REML), which converged with a REML criterion of 2353.8, indicating a good fit to the data.

In this analysis, random effects were included to account for variations across subjects not captured by the fixed effects in the model. The variance for the random intercepts associated with subjects was 0.002338, corresponding to a standard deviation of 0.04836. This relatively low value suggests minor variability in baseline accuracy across individuals. Additionally, the residual variance was 0.194787, with a standard deviation of 0.44135, indicating that a substantial amount of variability in accuracy remains unexplained by the model.

For the fixed effects, the intercept, representing the baseline accuracy in the random priming condition, was significantly positive at 0.264 with a standard error of 0.015, reflecting a substantial level of accuracy and yielding a *t*-value of 17.520. This indicates a significant baseline accuracy level (*M* = 0.264, SE = 0.0151, CI = 0.234–0.293). The coefficient for perceptual priming was 0.013, with a standard error of 0.020, resulting in a *t*-value of 0.635. This suggests that the change in accuracy associated with the perceptual priming (*M* = 0.277, SE = 0.0153, CI = 0.246–0.307), compared to the baseline, is not statistically significant. The negative correlation of −0.656 between the intercept and the effect of perceptual priming suggests an inverse relationship; however, given that the effect of the perceptual priming is not significant, this correlation primarily reflects the variance structure of the model rather than a meaningful interaction between these terms.

The linear mixed model analysis was also conducted to explore how different perceptual priming influences participants’ confidence in choices without explicit knowledge. Using restricted maximum likelihood for the estimation, the model achieved convergence with a REML criterion of 11314.4, suggesting a good fit with the dataset. The random effects structure of the model revealed significant variability among subjects, with a standard deviation of 1.429 for the subject-specific intercepts. This indicates notable differences in how individuals generally rated their confidence. The residual variance, with a standard deviation of 1.321, points to a considerable amount of response variability not explained by the differences in priming or individual baseline tendencies.

Regarding fixed effects, the confidence ratings for incoherent tetrads primed with random words, represented by the intercept, were significantly positive at 2.496, with a robust *t*-value of 17.292 (*M* = 0.2.50, SE = 0.144, CI = 2.21–2.78). The effect of the random priming of coherent tetrads was small and not statistically significant, with an estimate of 0.032 and a *t*-value of 0.563, indicating that this condition does not alter response levels in a meaningful way compared to the baseline (*M* = 2.53, SE = 0.144, CI = 2.24–2.81). Conversely, the perceptual priming condition showed a more substantial positive effect on responses, with an estimate of 0.200 and a *t*-value of 3.487, suggesting that this priming notably enhances participant responses (*M* = 2.70, SE = 0.145, CI = 2.41–2.98). The correlations between the fixed effects were relatively low, indicating that while there is some interaction between the different block types’ effects, each has a distinct impact on the responses. This nuanced view highlights that while some priming conditions can significantly alter responses, others might not have such a pronounced effect.

Similarly, to Study 1, we ran the LMM analysis to assess how perceptual priming, accuracy of choices, and their interaction influence participants’ confidence ratings. The model was fitted using restricted maximum likelihood, achieving convergence with a REML criterion 1460.4. This model noted substantial individual variability in baseline confidence levels, as evidenced by a standard deviation of 1.6020 for the random intercepts. This substantial variation suggests that individual differences significantly affect confidence ratings. Additionally, the residual variance was 0.9339, with a standard deviation of 0.9664, indicating that considerable variability in confidence remains unexplained by the factors included in the model.

Regarding fixed effects, the baseline confidence, representing random priming of coherent tetrads, was significantly high at 3.2885, with a robust *t*-value of 18.227, indicating a moderate confidence level. The impact of the coherent perceptual priming on confidence was minor and not statistically significant, with an estimate of 0.1102 and a *t*-value of 0.835, suggesting that this block type does not substantially alter confidence levels compared to the baseline.

In contrast, the accuracy of choices had a significant positive effect on confidence, with an estimate of 0.6749 and a *t*-value of 5.108, indicating that correct responses are associated with higher confidence. Generally, participants rated their confidence in correct choices (*M* = 3.96, *SE* = 0.18, *CI* = 3.61–4.32 for random priming, and *M* = 4.27, *SE* = 0.18, *CI* = 3.91–4.63 for coherent perceptual priming) higher than incorrect choices (*M* = 3.29, *SE* = 0.18, *CI* = 2.93–3.64 for random priming, and *M* = 3.40, *SE* = 0.18, *CI* = 3.04–3.75 for coherent priming).

The interaction between priming and choice accuracy showed an estimate of 0.1963 and a *t*-value of 1.051, pointing to a marginally significant effect. This suggests that the influence of accuracy on confidence might slightly vary depending on the block type, although this effect was not pronounced.

### Discussion

In Study 2, the objective was to investigate the impact of perceptual priming on the intuitive detection of semantic coherence, expanding upon the findings of Study 1, which focused on semantic priming. This study aimed to discern whether perceptual elements of priming could similarly influence cognitive processes involved in linguistic intuition. Contrary to the significant effects observed with semantic priming in Study 1, the results from Study 2 revealed that perceptual priming exerts a more subtle influence on the accuracy and confidence of intuitive judgments.

The investigation into perceptual priming demonstrates that, while it does not significantly alter the accuracy of identifying semantic incoherences, it notably impacts participants’ confidence in their choices. This suggests a differentiated role of perceptual versus semantic priming in cognitive processing, with perceptual priming primarily affecting the confidence with which judgments are made rather than their correctness.

These findings contribute to the broader discourse on cognitive processing in linguistic tasks, suggesting that the nature of the priming influences the mechanisms underlying intuitive judgments. The subtle yet significant role of perceptual priming in shaping confidence without markedly affecting accuracy underscores the complexity of cognitive processes governing linguistic intuition. This research thereby extends the understanding of how different types of priming influence the cognitive underpinnings of intuition in linguistic tasks, highlighting the nuanced interplay between perceptual and semantic factors in shaping both the accuracy and confidence of judgments.

## General discussion

The findings of the current studies resonate with the theoretical model outlined in the papers (Topolinski and Strack, 2009a; Sweklej et al., 2014), which emphasize the role of processing fluency, mood, and affect in guiding intuitive judgments of semantic coherence. The present results extend these models by differentiating the effects of semantic and perceptual priming, suggesting that semantic priming aligns more closely with the proposed mechanisms where enhanced processing fluency and positive affective responses facilitate more accurate and confident coherence judgments.

The Associative-Propositional Evaluation (APE) model by Gawronski and Bodenhausen (2011) posits that evaluations can arise from two distinct processes: associative and propositional. Associative processes involve automatic activations of mental associations, whereas propositional processes involve validating these associations against other information. This dual-process framework allows for the simultaneous operation of both implicit (associative) and explicit (propositional) evaluations, explaining complex cognitive phenomena such as intuition and judgment (Gawronski and Bodenhausen, 2006).

Study 1 examined the effects of semantic priming on intuitive judgments of semantic coherence. The results demonstrated that semantic priming significantly enhances the accuracy and confidence of participants’ judgments. From the perspective of the APE model, these findings can be interpreted as follows: The exposure to semantically coherent primes likely activated relevant associations in participants’ memory networks, facilitating quicker and more accurate identification of incoherent elements. This aligns with the notion that associative processes are automatic and influence implicit evaluations (Gawronski and Bodenhausen, 2011). Additionally, the increased confidence observed in participants’ judgments suggests that the activated associations were further validated through propositional reasoning, supporting the explicit evaluation of coherence. The dual influence of these processes illustrates how semantic priming can engage both automatic and reflective cognitive mechanisms to enhance intuitive decision-making.

Study 2 focused on perceptual priming and its impact on semantic coherence judgments. Unlike semantic priming, perceptual priming did not significantly improve accuracy but did increase confidence in participants’ judgments. According to the APE model, perceptual priming may not activate semantic associations as effectively as semantic priming, leading to less pronounced improvements in accuracy. This suggests that associative processes are more effectively triggered by semantically relevant stimuli. Despite the lack of significant improvement in accuracy, the increase in confidence indicates that perceptual fluency might still influence propositional validation processes. Participants may have interpreted the ease of perceptual processing as a cue for coherence, thus impacting their explicit evaluations.

The findings from both studies underscore the nuanced roles of associative and propositional processes in shaping intuitive judgments. Semantic priming effectively leverages both processes, enhancing accuracy and confidence intuitive choices. In contrast, perceptual priming primarily influences explicit metacognitive assessment of one’s confidence without significantly affecting accuracy. These differential effects highlight the importance of considering both types of processes in understanding how priming influences intuitive judgments. Future research should explore additional dimensions of priming, such as emotional or contextual priming, and their interactions with associative and propositional processes. This approach will further elucidate the complex interplay between automatic and reflective cognitive mechanisms in shaping intuition and judgment.

Despite these contributions, the current manuscript is not without limitations. One such limitation includes the relatively controlled experimental conditions that may not fully capture the complexity of intuitive judgments in naturalistic settings. Additionally, the direct comparison between semantic and perceptual priming effects was constrained to specific types of priming, potentially overlooking other relevant dimensions of priming that could influence intuition in linguistic tasks. Additionally, the exclusive focus on shorter linguistic structures (triads and tetrads) may not fully encapsulate the complexity of semantic coherence in natural language contexts. Future research should consider using longer semantic contexts and exploring variations in prime presentation duration to enhance the applicability of findings to real-world linguistic tasks. For example, the flexibility and context-dependency of word semantics are emphasized in linguistic studies (Evans, 2006). The meaning of words can vary significantly based on the context in which they are used, highlighting the importance of considering the broader linguistic context in semantic analysis. Research has also demonstrated that contextual factors influence semantic representations during language comprehension (Deniz et al., 2021).

The current study’s exclusive use of triads and tetrads may not fully capture the complexity inherent in natural language processing, which often involves more extended and contextually rich linguistic structures. Research indicates that longer semantic contexts can provide a more comprehensive understanding of semantic coherence by engaging more elaborate neural and cognitive mechanisms (Whiting et al., 2017). By incorporating longer sentences or paragraphs as stimuli, future studies could enhance the ecological validity of the findings and offer deeper insights into how semantic coherence is processed in real-world linguistic tasks.

Additionally, varying the duration of prime presentations can offer valuable insights into the gradience of priming effects. Studies have shown that different prime durations can modulate the strength and nature of priming effects, with longer durations potentially increasing the activation of the prime and its influence on the target (Dijkstra et al., 2023). By exploring a range of prime durations, future research can better understand the temporal dynamics of semantic and perceptual priming, thereby providing a more nuanced view of how these mechanisms influence intuitive judgments. These modifications would address the current limitations and contribute to a richer understanding of the cognitive processes underlying semantic coherence judgments.

Future research directions could address these limitations and further elaborate on the theoretical model of intuitive semantic coherence judgments. Investigations could explore additional forms of priming, such as contextual or emotional priming, and their interaction with semantic and perceptual elements. Moreover, examining the role of individual differences, such as cognitive style or linguistic proficiency, could provide deeper insights into the variability of intuitive judgments. Lastly, employing naturalistic tasks and settings may enhance the ecological validity of the findings, offering a more comprehensive understanding of how these cognitive processes operate in everyday linguistic judgments.

In sum, the current studies contribute to a better understanding of intuitive semantic coherence judgments, highlighting the distinct roles of semantic and perceptual priming. By situating these findings within the broader theoretical (dual-process theories) and empirical context (a novel task to study intuitive judgments of coherence), we hope this research addresses critical aspects of cognitive processing in linguistic intuition and opens avenues for future explorations that could further illuminate the intricate mechanisms underlying human cognition and language comprehension.

## Data availability statement

The raw data supporting the conclusions of this article will be made available by the authors, without undue reservation.

## Ethics statement

The studies involving humans were approved by Ethics Committee, SWPS University. The studies were conducted in accordance with the local legislation and institutional requirements. The participants provided their written informed consent to participate in this study.

## Author contributions

JS: Writing – review & editing, Writing – original draft, Validation, Supervision, Resources, Project administration, Methodology, Investigation, Funding acquisition, Conceptualization. RB: Writing – review & editing, Writing – original draft, Software, Resources, Methodology, Investigation, Conceptualization.

## Footnotes

^1^It is worth noting that degraded presentation of semantic or perceptual primes does not ensure lack of awareness of those primes. The lack of awareness is not a precondition of priming effect that may occur both under subliminal and optimal condition. It does not also directly address the issue of implicit or explicit processes under priming effects since those processes operate at the response level as well. However, the issue of how awareness of primes influences intuitive judgments of semantic coherence is definitely worth its own empirical investigation.

^2^However, some of the further LMM analyses presented a singular model fit suggesting too little variance in random slopes and/or intercept. In such cases we applied a standard ANOVA or *t*-test approaches.
