# Supplementary material for: Semantic versus perceptual priming: dissecting their impact on intuitive judgments of semantic coherence
Source: Front Psychol. 2024 Jun 25;15:1406811. doi: 10.3389/fpsyg.2024.1406811 (PMC11231393; doi:10.3389/fpsyg.2024.1406811)
Supplement: Supplementary file 1 [file Table_1.docx]

Table 1. Solvable Tetrads used in Study 1 and 2.

| LP | WORD1 | WORD2 | WORD3 | WORD4 - unrelated | SOLUTION/PRIME |
| --- | --- | --- | --- | --- | --- |
| 1 | duck | fold | dollar | wise | bill |
| 2 | sleeping | bean | trash | roll | bag |
| 3 | dew | comb | bee | fork | honey |
| 4 | night | wrist | stop | man | watch |
| 5 | loser | throat | spot | grass | sore |
| 6 | cane | daddy | plum | cry | sugar |
| 7 | preserve | ranger | tropical | pea | forest |
| 8 | aid | rubber | wagon | fight | band |
| 9 | dream | break | light | dive | day |
| 10 | safety | cushion | point | shock | pin |
| 11 | worm | shelf | end | oil | book |
| 12 | flake | mobile | cone | foul | snow |
| 13 | fish | mine | rush | test | gold |
| 14 | cracker | fly | fighter | spoon | fire |
| 15 | measure | worm | video | note | tape |
| 16 | high | district | house | pet | school |
| 17 | fox | man | peep | board | hole |
| 18 | boot | summer | ground | illness | camp |
| 19 | date | alley | fold | mail | blind |
| 20 | peach | arm | tar | home | pit |
| 21 | print | berry | bird | end | blue |
| 22 | pie | luck | belly | way | pot |
| 23 | light | birthday | stick | stick | candle |
| 24 | cadet | capsule | ship | line | space |
| 25 | fly | clip | wall | bump | paper |
| 26 | sense | courtesy | place | mate | common |
| 27 | opera | hand | dish | self | soap |
| 28 | hound | pressure | shot | nose | blood |
| 29 | main | sweeper | light | artist | street |
| 30 | carpet | alert | ink | forward | red |
| 31 | horse | human | drag | back | race |
| 32 | wheel | hand | shopping | bottom | cart |
| 33 | river | note | account | child | bank |
| 34 | french | car | shoe | control | horn |
| 35 | water | mine | shaker | land | salt |
| 36 | sage | paint | hair | shadow | brush |
| 37 | mouse | bear | sand | cast | trap |
| 38 | age | mile | sand | reading | stone |
| 39 | force | line | mail | over | air |
| 40 | political | surprise | line | hungry | party |
