# Supplementary material for: Semantic versus perceptual priming: dissecting their impact on intuitive judgments of semantic coherence
Source: Front Psychol. 2024 Jun 25;15:1406811. doi: 10.3389/fpsyg.2024.1406811 (PMC11231393; doi:10.3389/fpsyg.2024.1406811)
Supplement: Supplementary file 2 [file Table_2.docx]

Table 2. Unsolvable Tetrads used in Study 1 and 2.

| LP | WORD1 | WORD2 | WORD3 | WORD4 | NONWORD |
| --- | --- | --- | --- | --- | --- |
| 1 | bottom | hammer | line | call | baga |
| 2 | control | nuclear | wear | shower | guho |
| 3 | bus | officer | tail | pen | gani |
| 4 | light | hold | secret | club | fika |
| 5 | front | rope | album | under | keni |
| 6 | bean | home | first | box | polu |
| 7 | chart | wagon | start | bow | nida |
| 8 | egg | cat | stomach | figure | huce |
| 9 | maker | type | dating | tow | mawi |
| 10 | curve | pine | hunter | station | wiko |
| 11 | work | health | less | petty | lawy |
| 12 | place | time | goal | gate | qrom |
| 13 | flush | keg | heart | fall | fyta |
| 14 | bar | eight | stool | post | pire |
| 15 | glue | dress | stick | cold | muni |
| 16 | board | baby | cake | top | bida |
| 17 | plant | piece | phone | coffee | tevq |
| 18 | shave | blank | down | full | pulv |
| 19 | arm | teeth | room | powder | rysa |
| 20 | ground | sandwich | master | foot | kopx |
| 21 | fruit | cover | mate | acid | vysi |
| 22 | side | fence | business | base | nyre |
| 23 | shoes | rain | flower | head | ceda |
| 24 | runner | knife | signal | sun | jufa |
| 25 | hatch | pike | engine | game | arku |
| 26 | hand | tail | picker | family | xifo |
| 27 | service | fur | nelson | false | segz |
| 28 | cloth | trip | golf | care | soju |
| 29 | chain | cross | cap | dog | zure |
| 30 | king | break | bed | apple | motq |
| 31 | blade | wet | sauce | turn | bidk |
| 32 | ground | tomato | war | sweet | podb |
| 33 | attorney | tank | hat | writer | jufe |
| 34 | dark | off | tie | steam | odgi |
| 35 | shell | tooth | larceny | coat | efbu |
| 36 | order | guy | screen | check | ocxa |
| 37 | line | iron | hot | field | aghe |
| 38 | step | catcher | flood | sick | butr |
| 39 | scan | office | radio | cherry | pofq |
| 40 | stone | cut | pal | suit | hitr |
